# New insights into the crosstalk between endocannabinoids and sphingosine-1-phosphate

**DOI:** 10.1016/j.jbc.2025.110781

**Published:** 2025-09-29

**Authors:** Cinzia Rapino, Sara Standoli, Francesca Cencetti, Paola Bruni, Sergio Oddi, Mauro Maccarrone

**Affiliations:** 1 Department of Veterinary Medicine, University of Teramo, Teramo, Italy; 2 Department of Experimental and Clinical Biomedical Sciences “Mario Serio”, University of Florence, Florence, Italy; 3 Laboratory of Lipid Neurochemistry, European Center for Brain Research, IRCCS Santa Lucia Foundation of Rome, Rome, Italy; 4 Department of Biotechnological and Applied Clinical Sciences, University of L’Aquila, L’Aquila, Italy

**Keywords:** endocannabinoids, anandamide, 2-arachidonoylglycerol, sphingosine-1-phosphate, cannabidiol, crosstalk, GPCRs, TRPV1, PPARγ

## Abstract

This review aims at highlighting the interplay between the endocannabinoids (eCBs) anandamide and 2-arachidonoylglycerol and sphingosine-1-phosphate (S1P) signaling. The eCBs and S1P are bioactive compounds that exemplify a paradigm of crosstalk among lipid signals, with profound implications for physiological processes and disease pathogenesis. Crosscommunication between eCBs and S1P occurs through multiple mechanisms: (i) receptor heterodimerization and coregulation, (ii) mutual metabolic modulation, and (iii) integrated regulation of downstream effectors. The latter emerged as a key mechanism underlying the bidirectional interactions between eCBs and S1P, with functional overlaps that regulate several processes, including inflammation, vascular function, and neuronal activity. In addition, cannabis-derived compounds (such as cannabidiol) can influence eCBs and S1P signaling, calling for further research into their therapeutic exploitation. Overall, the dynamic interplay between endogenous eCBs and S1P—as well as with exogenous cannabidiol—described here offers a compelling example of the complexity of interactions among bioactive lipids. A deeper mechanistic understanding of these relationships could pave the way to novel strategies in drug design and development, emphasizing the importance of integrated approaches in the study of bioactive lipid biochemistry.

## Bioactive lipids: Endocannabinoids *versus* sphingosine-1-phosphate

Bioactive lipids—particularly endocannabinoids (eCBs) and sphingosine-1-phosphate (S1P)—act as cellular messengers with highly dynamic transport and trafficking mechanisms that drive their signaling activity (1, 2). The major eCBs are anandamide (*N*-arachidonoylethanolamine [AEA]) and 2-arachidonoylglycerol (2-AG), which possess multiple biological functions (1, 3, 4). AEA and 2-AG are, respectively, an *N*-acylethanolamine (NAE) and a glycerol ester formed through condensation with the carboxylic group of arachidonic acid (AA) (5, 6). Both eCBs serve as key homeostatic regulators throughout the body and play vital roles in numerous physiological functions, such as brain development, cell survival, neurotransmission, pain sensation, immune response, energy homeostasis, bone metabolism, and vascular tone, just to list a few (1, 7).

S1P is an important bioactive sphingolipid with a distinctive molecular structure typically characterized by an 18-carbon sphingosine backbone and a phosphate group esterified to the first hydroxyl position (8, 9). However, the sphingoid base chain length can vary significantly, ranging from 12 to 22 carbons (10). S1P is present in biological fluids and is locally produced by most cell types and tissues, with particular relevance in the central nervous system (CNS), immune system, and vascular system (11).

Despite their different origins, eCBs and S1P share key molecular features. Indeed, they are both primarily produced “on demand” upon specific cellular stimuli (12, 13); in addition, eCBs—but not S1P—can also be stored in cytosolic organelles such as adiposomes (14, 15). Both eCBs and S1P require specific carriers for transmembrane transport (in and out) and trafficking (1, 16, 17) and usually act as local mediators with short-range paracrine action because of rapid degradation by specific enzymes (1, 16, 17). Furthermore, eCBs and S1P are ligands at different G-protein–coupled receptors (GPCRs) (18, 19) with putative crosstalk capabilities (20, 21, 22).

In this review, we will critically discuss the points of contact and intersection between eCBs and S1P signaling, at the molecular and functional levels.

## A common strategy of metabolic enzymes, receptors, and transporters

The metabolic routes of eCBs and S1P reveal intriguing commonalities, despite the distinct chemical nature of these lipids. The endogenous levels of both classes are tightly controlled by distinct biosynthetic and catabolic enzymes, which in the case of AEA and 2-AG lead to different *in vivo* concentrations (higher for 2-AG than for AEA), and hence to distinct receptor activation and signaling thereof (23).

The biosynthesis of AEA and 2-AG occurs mainly through the action of *N*-acyl-phosphatidylethanolamine–specific phospholipase D (NAPE-PLD) (24) and diacylglycerol lipase α and β isoforms (25, 26, 27), respectively. However, studies performed in NAPE-PLD knockout mice have revealed additional pathways (28), with an apparent redundancy that warrants AEA production even when the primary NAPE-PLD–dependent pathway is compromised.

AEA is primarily degraded by fatty acid amide hydrolase (FAAH) into AA and ethanolamine (EtNH_2_) (29), whereas 2-AG is metabolized by monoacylglycerol lipase (MAGL) into AA and glycerol (30). Alternatively, AEA can undergo oxygenation *via* cyclooxygenase-2 (COX-2), forming prostaglandin-H_2_-EtNH_2_ (31, 32), or it can be transformed by lipoxygenase (LOX) isozymes—5-LOX, 12-LOX, and 15-LOX—into hosphor derivatives like 11-HETE-EA, 12-HETE-EA, and 15-HETE-EA, respectively (33, 34). Finally, cytochrome P450 (CYP) enzymes can epoxygenate AEA to form epoxyeicosatrienoyl-ethanolamide (35, 36).

Much alike AEA, 2-AG can be oxygenated by COX-2, leading to PG-glycerol (37, 38) and by LOX isozymes that form hosphor derivatives such as 12-HETE-G, 15-HETE-G, and eoxin derivatives (39, 40, 41). Epoxygenation of 2-AG by CYP at various positions results in different epoxyeicosatrienoyl-glycerols (42, 43). These oxidative pathways represent critical control points for eCB signaling duration and can lead to new metabolites endowed with their own biological activity (44).

As for S1P, it is synthesized by ATP-dependent phosphorylation of sphingosine, catalyzed by sphingosine kinases 1 (SphK1) and 2 (SphK2), which have distinct subcellular localizations: SphK1 is primarily in the cytoplasm and cell membranes, whereas SphK2 is present mainly in mitochondria, nucleus, and endoplasmic reticulum (45, 46, 47). The SphK substrate sphingosine is, in turn, produced by ceramide hydrolysis driven by ceramidases (48). The catabolic pathways of cellular S1P involve S1P phosphatase–dependent dephosphorylation and irreversible cleavage into hexadecenal and EtNH_2_ phosphate *via* S1P lyase, which represents the only exit point for sphingolipid degradation (49). Extracellular S1P can also be degraded by lysophospholipid phosphatase 3 into sphingosine, which can then be taken up by cells for further metabolism (50).

Both eCBs and S1P primarily signal through distinct families of GPCRs. In particular, AEA and 2-AG target cannabinoid receptors 1 and 2 (CB_1_ and CB_2_), whereas S1P activates five distinct receptors (S1PR_1–5_) with both systems exhibiting overlapping anatomical distributions in the CNS and peripheral tissues (51, 52, 53, 54). In addition to canonical CB_1_ and CB_2_ receptors, eCBs can bind to other GPCRs like GPR55, as well as non-GPCRs such as transient receptor potential vanilloid-1 (TRPV1) channel and nuclear peroxisome proliferator–activated receptors (PPARs) α, γ, and δ (20, 55, 56).

The biological activity of eCBs and S1P—both acting as intracellular messengers and extracellular agonists—is subjected to spatiotemporal regulation, which is tightly dependent on metabolic enzymes and transport/trafficking mechanisms (14, 57, 58, 59). For instance, in the case of S1P, its metabolism is finely regulated by diverse stimuli, including growth factors, cytokines, hormones, and neurotransmitters (57, 59), and the tissue-specific expression of S1P_1–5_ receptors contributes to the selectivity of intracellular signaling pathways triggered thereof (60). Much alike eCBs (61, 62), the rapid metabolism of S1P is critical to maintain its endogenous tone and hence signaling pathways.

Of note, S1P is not the only bioactive sphingolipid. Indeed, ceramide—considered the central hub of the sphingolipid pathway—has opposite effects compared with S1P: it induces apoptosis, whereas S1P promotes cell survival and proliferation (63). Remarkably, ceramide metabolism is closely interconnected with that of S1P and is regarded as the “sphingolipid rheostat” that determines cell fate (64). Due to its polar nature, intracellularly produced S1P must flip to the outer layer of plasma membrane in order to interact with its receptors (the so-called inside–out signaling) or to be released into the bloodstream (65). In this context, it should be recalled that S1P levels significantly vary between different body districts and may be altered under disease conditions (11).

Although eCBs and S1P are primarily produced inside the cell and then exported outside in response to various stimuli, emerging evidence demonstrates that both lipids can also be packaged into and transported by extracellular vesicles (Evs) (66, 67). Indeed, Evs serve as carriers that protect these hydrophobic signals from degradation and facilitate their transport across aqueous environments to distant cellular targets. The packaging of eCBs in microvesicles has been demonstrated in microglial cells, where AEA localizes on vesicle surfaces and remains biologically active, that is, capable of binding to and activating presynaptic CB_1_ receptors to modulate synaptic transmission (66, 68). Similarly, S1P—and in some cases even its biosynthetic enzymes—have been detected within Evs released from various cell types, such as hepatocarcinoma cells, endothelial cells, and pancreatic β-cells (67, 69, 70, 71). The vesicular transport of eCBs and S1P not only extends their signaling range beyond the production sites but also provides a mechanism that enables navigation of these lipids through aqueous extracellular environments to reach their ultimate targets.

**Figure 1:**
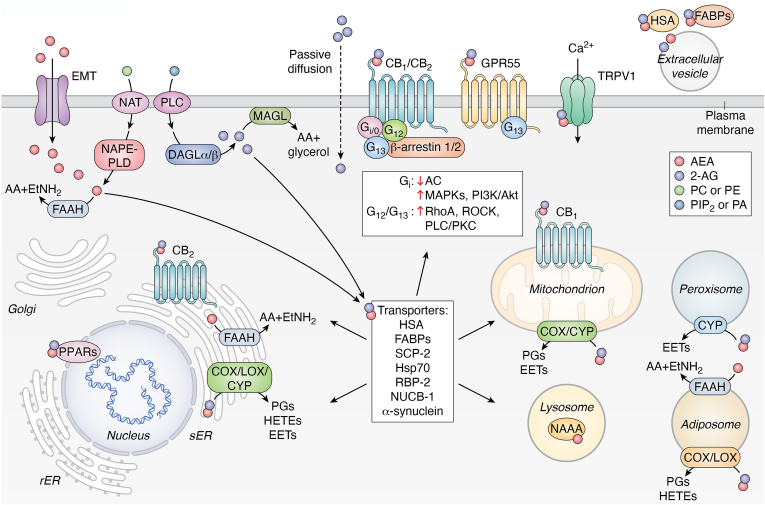
**Diagram of the complete endocannabinoid system, illustrating its receptors, biosynthetic and degradative enzymes, trafficking and transporters.** AEA, anandamide; 2-AG, 2-arachidonoylglycerol; α-Syn, alpha-synuclein; CB_1,_ cannabinoid receptor type 1; CB_2,_ cannabinoid receptor type 2; COX, cyclooxygenase; CYP, cytochrome P450; DAGLα/β, diacylglycerol lipase α/β; EET, epoxyeicosatrienoic acid; EMT, endocannabinoid membrane transporter; FAAH, fatty acid amide hydrolase; FABP, fatty acid binding protein; GPCR, G-protein–coupled receptor; GPR55, G-protein–coupled receptor 55; HETE, hydroxyeicosatetraenoic acid; HSP70, heat shock protein 70; LOX, lipoxygenase; MAGL, monoacylglycerol lipase; NAAA, *N*-acylethanolamine acid amidase; NAPE-PLD, *N*-acyl-phosphatidylethanolamine-specific phospholipase D; NAT, *N*-acyltransferase; NUCB1, nucleobindin-1; PA, phosphatidic acid; PC, phosphatidylcholine; PE, phosphatidylethanolamine; PG, prostaglandin; PIP2, phosphatidylinositol 4,5-bisphosphate; PLC, phospholipase C; PPAR, peroxisome proliferator–activated receptor; RBP2, retinol-binding protein 2; SCP-2, sterol carrier protein 2; TRPV1, transient receptor potential vanilloid 1. Image provided by Servier Medical Art (https://smart.servier.com/), licensed under CC BY 4.0 (https://creativecommons.org/licenses/by/4.0/).

**Figure 2:**
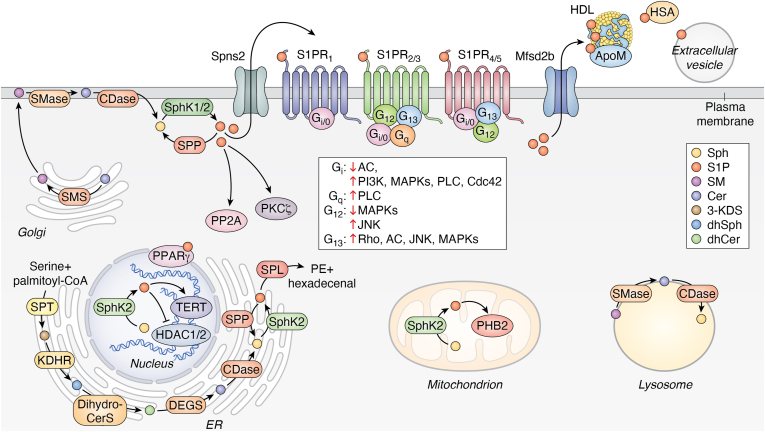
**Diagram of the complete sphingosine-1-phosphate (S1P) system, illustrating its receptors, biosynthetic and degradative enzymes, trafficking and transporters.** ApoM, apolipoprotein M; Cer, ceramide; CDase, ceramidase; DEGS, dihydroceramide desaturase; Dihydro-CerS, dihydroceramide synthase; GSL, glycosphingolipid; HDAC1/2, histone deacetylase 1 and 2; HDL, high-density lipoprotein; HSA, human serum albumin; KDHR, ketodihydrosphingosine reductase; Mfsd2b, major facilitator superfamily domain–containing 2B transporter; PE, phosphoethanolamine; PHB2, prohibitin-2; PKCζ, PKC zeta; PP2A, protein phosphatase 2A; PPARγ, peroxisome proliferator–activated receptor γ; S1P1–5, sphingosine-1-phosphate receptor 1–5; SM, sphingomyelin; SMase, sphingomyelinase; SMS, sphingomyelin synthase; Sph, sphingosine; SphK1, sphingosine kinase 1; SphK2, sphingosine kinase 2; SPL, sphingosine-1-phosphate lyase; Spns2, spinster homolog 2 transporter; SPP, sphingosine-1-phosphate phosphatase; SPT, serine palmitoyltransferase; TERT, telomerase reverse transcriptase. Image provided by Servier Medical Art (https://smart.servier.com/), licensed under CC BY 4.0 (https://creativecommons.org/licenses/by/4.0/).

Further details of eCB and S1P systems—including metabolic enzymes, receptors, transporters, and trafficking mechanisms—are summarized in Box 1 and Box 2, respectively (Figs. 1 and 2).

Box 1 **The eCB system**

The eCB system represents a complex network of signaling pathways primarily mediated by endogenous ligands known as eCBs, including AEA and 2-AG (1). AEA biosynthesis begins with the metabolism of AA-containing NAPEs, which are derived from the acylation of the amine group of phosphatidylethanolamines by both Ca^2+^-dependent and –independent *N*-acyltransferases, including cytosolic phospholipase A2ε (113).

Several biosynthetic routes contribute to AEA and other NAE formation. In the classical pathway, NAPE-PLD hydrolyzes NAPEs (24, 114), whereas alternative pathways involve phospholipase C (PLC)-hydrolyzing NAPEs to generate hosphor-AEA (115), which is subsequently dephosphorylated by phosphatases, including PTPN22 and SHIP1 (115, 116, 117). In addition, PLA_2_ generates lyso-NAPE, which is further hydrolyzed by lysophospholipase D (117, 118).

Once biosynthesized, AEA can undergo degradation or be transformed by oxygenation. Known enzymes of AEA hydrolysis include (1) FAAH, which hydrolyzes AEA (but also other NAEs) into AA and EtNH_2_, controlling the endogenous AEA tone (29, 119); (2) the lysosomal *N*-acylethanolamine acid amidase, which preferentially processes saturated NAEs, such as *N*-palmitoyl-EtNH_2_ (120, 121).

2-AG biosynthesis originates from AA-containing diacylglycerols (DAGs), typically derived from PLC-β hydrolysis of phosphatidylinositol 4,5-bisphosphate or from phosphatidic acid hydrolysis by phosphatidic acid phosphohydrolase (122). Subsequently, Ca^2+^-dependent PLC or DAG lipases (α and β) further hydrolyze DAG to release 2-AG (25, 26, 27). 2-AG is degraded mainly by MAGL (30) or by α,β-hydrolase domain–containing proteins, ABHD12 and ABHD6, responsible for approximately 9% and 4%, respectively, or by carboxylesterases (CES1 and CES2), producing fatty acids and glycerol (123, 124).

The biological activity of AEA and 2-AG is primarily mediated through interactions with cannabinoid receptors type 1 and 2 (CB_1_ and CB_2_), which belong to the GPCR superfamily (125). Upon activation, CB_1/2_ receptors modulate diverse intracellular signaling cascades, including those involving cAMP, PI3K, and MAPK. This results in varied cellular responses, such as alterations in cell motility, survival, proliferation, and neurotransmitter release (126, 127).

Specifically, CB_1_ is predominantly expressed in the CNS, including regions such as the neocortex, hippocampus, basal ganglia, cerebellum, and brainstem, whereas CB_2_ is primarily found in immune cells and peripheral tissues, with lower levels present in the CNS, especially in microglial cells and neurons of the brainstem and spinal cord (22, 128).

In addition to CB_1_ and CB_2_, AEA and 2-AG can also activate nonclassical cannabinoid receptors, such as GPR55 (55, 129). GPR55 is implicated in signaling pathways related to calcium release and Ras homolog gene family member A activation, affecting cell proliferation and migration (55, 127, 130). Furthermore, eCBs, especially AEA and 2-AG, interact with the TRPV1 receptor, a cation channel involved in nociception and thermoregulation. eCBs bind to the same vanilloid-binding site as capsaicin but with distinct configurations that reduce their partial agonist efficacy compared with capsaicin (20, 21, 56, 131).

Despite their preference for GPCRs, both AEA and 2-AG can also activate PPARs, which are nuclear receptors that modulate gene expression by binding to DNA peroxisome proliferator response elements. These receptors have three isoforms: PPARα, PPARγ, and PPARδ (74, 132, 133). AEA and 2-AG primarily activate PPARα and PPARγ, thereby influencing lipid metabolism, energy homeostasis, and inflammatory responses (74). Notably, PPARα activation by eCBs plays a significant role in hepatic lipid metabolism, whereas PPARγ modulates adipogenesis and glucose metabolism (133).

As uncharged lipids, eCBs can cross the membrane bilayer through passive diffusion (134) or facilitated diffusion *via* a putative transmembrane carrier termed eCB membrane transport (135, 136), which acts bidirectionally (135). Once inside the cell, intracellular binding proteins play crucial roles in not only eCB trafficking, particularly fatty acid–binding proteins, but also retinol-binding protein 2, sterol carrier protein 2, nucleobindin-1, human serum albumin, and heat shock protein 70 for breakdown by the hydrolase FAAH, thereby terminating signal transduction and receptor signaling (137, 138, 139, 140, 141, 142, 143, 144).

Alternatively, eCBs can be transported to other cellular compartments, such as (i) mitochondria for oxidation by COX-2 or CYP (14, 36, 145) and potential activation of CB_1_ (146, 147); (ii) lysosomes for degradation by *N*-acylethanolamine acid amidase (121, 146); or (iii) the nucleus for PPAR activation (14, 74).

In addition to accumulation and sequestration in specific organelles, extracellular transport of eCBs is mediated not only by fatty acid–binding proteins but also by Evs and proteins, such as human serum albumin, contributing to the fine regulation of eCB activity (15, 68, 137, 142, 148). Furthermore, synucleins (α-Syn) can facilitate postsynaptical eCB release, with synuclein deletion blocking eCB-mediated synaptic plasticity (149). These intricate regulations allow eCBs to exert precise and context-dependent effects in maintaining homeostasis throughout the body, and when disrupted, can lead to pathological conditions. The different receptors (GPCR, nuclear receptors, and TRPV1), metabolic enzymes, trafficking elements, and transmembrane transport of eCBs are schematically depicted in Figure 1.

Box 2 **The S1P system**

S1P is a potent bioactive sphingolipid generated by the ATP-dependent phosphorylation of sphingosine catalyzed by two distinct enzymes, SphK1 and 2, which differ in subcellular localization, regulatory properties, and often exert divergent functions (8, 150). This sphingolipid was regarded for a long time as a mere metabolite in the catabolic pathway of sphingolipids, where the only exit route is represented by the cleavage of S1P into phosphoethanolamine and hexadecenal, catalyzed by S1P lyase (151). However, it is now well established that S1P formation is intimately connected to ceramide metabolism, which is considered the central hub of sphingolipid metabolism, and is also biologically active.

Ceramide can be synthesized *de novo* in the endoplasmic reticulum through a pathway initiated by serine palmitoyltransferase, which catalyzes the condensation of serine and palmitoyl-CoA to form 3-keto-dihydrosphingosine. This product is then reduced to dihydrosphingosine, acylated by a family of six (dihydro)ceramide synthases to dihydroceramide and finally converted into ceramide by ceramide desaturase (152). Ceramide serves as a precursor of glycosphingolipids and sphingomyelin, which are key components of biological membranes. In the synthesis of sphingomyelin, a phosphorylcholine group is transferred from phosphatidylcholine to ceramide by sphingomyelin synthase. Alternatively, ceramide coupling with a glucose or a galactose residue initiates glycosphingolipid synthesis, eventually followed by further glycosyltransferase reactions (152, 153). Notably, sphingosine, which serves as a product of sphingomyelin catabolism, is largely salvaged through reacylation by (dihydro)ceramide synthase, thus producing ceramide, playing a prominent role in maintaining the cellular ceramide levels (154). The main pathway responsible for S1P biosynthesis relies on sphingomyelin hydrolysis driven by sphingomyelinase, which releases ceramide that is further cleaved by ceramidase to produce sphingosine, which is then phosphorylated by SphK1/2 (8, 150, 152).

Besides the S1P lyase–directed irreversible breakdown, S1P can also be dephosphorylated to sphingosine by two specific S1P phosphatases, whereas lysophospholipid phosphatase 3 has a prominent role in its dephosphorylation in extracellular compartments (50, 155). Consistent with its function as a cellular mediator, S1P metabolism is strictly controlled: SphK1 and SphK2 are highly regulated at transcriptional, translational, and post-translational levels (45, 46, 156).

Once formed, S1P can either act as an intracellular signaling molecule or be transported extracellularly to exert its effects. Two specific transporters, Mfsd2b and Spns2, in red blood cells, platelets, and endothelial cells, respectively (17, 157, 158), ensure the maintenance of plasma levels of S1P, whereas Spns2 alone participates in S1P export in other cellular contexts where S1P acts through the so-called inside–out signaling mechanism (65).

While S1P levels in most animal tissues are typically in the nanomolar range because of its rapid turnover, its concentration in plasma is approximately 100 times higher. Notably, the significant concentration gradient of S1P between plasma and interstitial fluids is important for immune cell homing to lymphoid organs and regulating their egress into blood and lymph (49, 159, 160).

The amphipathic nature of S1P hinders its free state in the aqueous phase; indeed, more than 50% of plasma S1P is associated with high-density lipoprotein, whereas approximately 35% is bound to albumin, and the remainder is likely bound to other lipoproteins. ApoM, a component of high-density lipoprotein, is the unique high-affinity S1P-binding protein that preserves S1P from hydrolysis and regulates its function by facilitating interaction with its receptors, thus enhancing its biological functions (17, 161, 162, 163, 164, 165).

S1P, acting as S1P_1–5_ ligand, has emerged as a potent bioactive lipid mediator, orchestrating numerous cellular functions, including cell proliferation, survival, motility, and differentiation. S1P receptors transmit diverse intracellular signals depending on the coupled Gα subunits of heterotrimeric G proteins and the expression pattern of each receptor in a given cell (8, 166, 167).

S1P_1_ is ubiquitously expressed, with a prominent role in controlling endothelial integrity and immune cell trafficking. It couples exclusively with Gi proteins, activating PLC, Ras, PI3K, and inhibiting adenylyl cyclase (168). S1P_2_, which is abundantly expressed, interacts with G_i_, G_q_, and G_13_ proteins and negatively regulates cell motility by activating Rho and inhibiting Rac (73, 169). S1P_3_, sharing structural homology with cannabinoid receptors and also abundantly expressed, interacts with G_i,_ G_q_, and G_13_ proteins (54). It regulates cytoskeletal reorganization and promotes tissue fibrosis. S1P_4_ and S1P_5_ expression is more restricted, with a role in the immune and nervous systems, respectively (13, 54, 170).

S1P, initially discovered as an intracellular messenger, also acts by binding to intracellular protein targets. Indeed, S1P can function as a ligand for the nuclear receptor PPARγ (75), as detailed in the text. Moreover, S1P produced within the nucleus specifically binds to histone deacetylase-1 and -2, thereby inhibiting their enzymatic activity and consequently affecting gene expression, thus linking S1P to epigenetic regulation of gene expression (171). The mitochondrial protein prohibitin-2 is another intracellular target of S1P, important for oxidative phosphorylation (172). In addition, S1P has been shown to bind to human telomerase reverse transcriptase by allosterically mimicking protein phosphorylation, suggesting a role in regulating telomere integrity, which may contribute to delaying cellular senescence and promoting longevity (173). Furthermore, it has been recently reported that in red blood cells, S1P regulates glucose transport by binding and activating of the catalytic subunit of protein phosphatase 2A to reduce GLUT1 phosphorylation, cell surface localization, and glucose uptake (174). Finally, cellular S1P has been shown to directly activate PKCζ by relieving autoinhibitory constraints (175), thereby driving lipolysis in adipocytes (176).

Collectively, this comprehensive picture underscores the complexity of sphingolipid metabolism and signaling, with special emphasis on S1P, as summarized in Figure 2.

## Crosstalk between eCBs and S1P

Crosscommunication between eCB and S1P systems occurs through multiple mechanisms: (i) receptor heterodimerization and coregulation, (ii) mutual metabolic modulation, and (iii) integrated regulation of downstream effectors.

### Heterodimerization and coregulation

Heterodimerization (*i.e.*, the formation of a complex between two different receptor partners) and coregulation of distinct receptors play crucial roles in intracellular signaling (72, 73), with a particular impact on the interaction between S1P-binding and eCB-binding receptors, and hence on energy homeostasis, cancer progression, and pain modulation.

The interaction between CB_2_ and S1P_5_ receptors forms a regulatory checkpoint that limits tumor progression in glioblastoma cells, as revealed by bioluminescence resonance energy transfer analysis (73). Confocal microscopy confirmed colocalization and cointernalization into the cytoplasm upon agonist stimulation. Moreover, CB_2_ upregulated tumor progression genes like Ki67 and p21, but S1P_5_ costimulation attenuated these effects by 60% to 70%. Such a CB_2_–S1P_5_ receptor heterodimerization expands the functional diversity of both lipid signaling systems, enabling context-dependent modulation of cellular responses (72, 73). The heterodimerization of S1P_5_ with GPR55 represents another regulatory checkpoint that balances protumorigenic and antitumorigenic signals. Indeed, GPR55 and S1P_5_ form a heterodimer in colon cancer cells, which involves the transmembrane segments of S1P_5_ with a minimal contribution from its C-terminal region, as demonstrated by bioluminescence resonance energy transfer experiments (72). When activated independently, each receptor promotes extracellular signal–regulated kinase phosphorylation, upregulation of protumorigenic genes ATF3, HIF-1α, and Sp1, and thus colon cancer cell proliferation. Instead, simultaneous stimulation of both receptors triggers cointernalization of the heterodimer, ultimately suppressing tumor progression (72).

An example of receptor coregulation is the ability of both S1P and eCBs to interact with PPARγ through distinct molecular mechanisms, thus influencing gene regulation and therapeutic outcomes (74, 75). Parham *et al.* (75) showed that S1P directly interacts with PPARγ through its ligand-binding domain that involves histidine 323, as revealed by *in silico* docking and *in vitro* binding assays. At physiological concentrations, S1P stimulates recruitment of the PPARγ coactivator 1-β, leading to the formation of a transcriptional complex within endothelial cells (75). Accordingly, S1P activates PPARγ-dependent gene reporters, selectively modulating target genes by upregulating PPARγ coactivator 1-β and plasminogen activator inhibitor-1, while suppressing the expression of the scavenger receptor CD36. Importantly, the proangiogenic action of S1P, leading to *in vitro* tube formation, is PPARγ dependent and is blocked by PPARγ antagonists (75).

Notably, AEA and 2-AG can also bind directly to the ligand-binding domain of PPARγ, contributing to anti-inflammatory and neuroprotective effects (74). Moreover, 2-AG also interacts indirectly with PPARγ *via* its COX-2-generated metabolites (76). Further indirect mechanisms of eCB–S1P intersection include (i) PPARγ ligand (*e.g*., ciglitazone)-dependent inhibition of FAAH and subsequent increase of AEA levels that potentiate PPARγ activation (77) and (ii) synergistic effect of CB_1_–CB_2_ signaling with PPARγ pathways, to mediate neuroprotection and related metabolic effects (74, 78, 79).

### Mutual metabolic modulation

Communication between the eCB and S1P systems also occurs through regulation of their metabolic enzymes. In rat carotid arteries, AEA induces relaxation through a mechanism that involves CB_2_, which subsequently activates SphK1 to produce S1P. The latter, in turn, acts *via* S1P_3_ receptor in the vascular endothelium to promote vasodilation (80). Similarly, the activation of SphK1, but not of SphK2, and the engagement of S1P_1_ that is expressed at the level of vascular smooth muscle, is required for the reduction of systolic blood pressure exerted by AEA (81).

Moreover, in lipopolysaccharide (LPS)-stimulated BV2 microglia cells, transcriptional upregulation of both SphK1 and SphK2 is induced, contributing to increased production of proinflammatory cytokines like tumor necrosis factor α and interleukin-1β (22). In the same cells, inhibition of FAAH prevents LPS-induced SphK1 and SphK2 upregulation as well as cytokine production. In addition, activation of CB_2_ by the synthetic agonist JWH133 mimics the anti-inflammatory effects of FAAH inhibition, positioning SphK1 and SphK2 at the intersection between proinflammatory LPS and anti-inflammatory eCB signaling pathways in microglia (22).

Also, LOX isozymes, which metabolize eCBs into oxylipin derivatives like hydroxyeicosatetraenoic acids (82), are modulated by S1P (83, 84). For instance, S1P can reduce 5-LOX activity—particularly in immune cells like neutrophils and macrophages—through S1P_4_ engagement (83). In general, it should be noted that regulation of AA metabolism by S1P likely affects eCB tone and biological activity. Although the eCB–S1P crosstalk *via* AA is complex and still under investigation, several mechanistic links support its potential *in vivo* relevance. S1P can influence intracellular AA mobilization by activating calcium-independent and calcium-dependent phospholipases A_2_ (iPLA_2_ and cPLA_2_) (85). By inducing the expression of the AA dioxygenase COX-2, S1P triggers a metabolic competition that favors the fueling of prostaglandin production at the expense of eCB synthesis (86). Depending on the target tissue, such a switch of AA metabolism toward prostaglandins or eCBs may tip the balance between proinflammatory and anti-inflammatory effects or pronociceptive and antinociceptive effects (87). While direct experimental evidence linking S1P-mediated regulation of AA metabolism to changes in eCB levels is limited, the biochemical overlaps between S1P and eCB systems speak in favor of this hypothesis. Ongoing research in lipidomics and cell signaling has begun to uncover this intricate crosstalk, especially in contexts such as neurodegenerative diseases, cancer, chronic pain, and immune function (88, 89, 90).

A third mechanism of intersection between S1P and eCB signal transduction pathways involves the eCB-binding receptor TRPV1, which is also a key effector of S1P in several cellular contexts (91, 92, 93). In fact, S1P transactivates TRPV1 in primary sensory neurons to regulate nociception and pain. In particular, TRPV1 is coexpressed with S1P_3_ receptors in subsets of nociceptors, where S1P_3_ stimulation potentiates TRPV1 activity, overall leading to heat hypersensitivity and pain (91). Likewise, S1P-induced calcium influx in dorsal root ganglia neurons is partially abolished by TRPV1 antagonists, whereas *in vivo* studies show that TRPV1-knockout mice exhibit significantly reduced pain responses (*e.g.*, wiping and thermal hyperalgesia) after S1P injection (92). At the mechanistic level, S1P enhances TRPV1-mediated thermal pain sensitivity *via* a rapid G_αi_-coupled signaling pathway that involves PI3K, PKC, and p38 mitogen-activated protein kinase (MAPK) l (93).

Finally, in a recent study performed in murine C2C12 myoblasts, S1P treatment for 24 h significantly increased TRPV1 expression at both mRNA and protein levels, whereas reducing CB_2_ protein levels. The opposite regulation of TRPV1 and CB_2_ by S1P suggests a mechanism whereby S1P channels eCB signaling toward TRPV1-dependent pathways, which are critical for calcium influx and mitochondrial function (21). In the same C2C12 cells, TRPV1 activation by the stable AEA analog methanandamide (mAEA) increases mitochondrial membrane potential (ΔΨ_m_) and upregulates PPARγ coactivator 1-α, a key regulator of mitochondrial biogenesis. Of note, treatment with S1P counteracts these effects of mAEA, likely through TRPV1 desensitization or altered calcium flux (21). The interplay between mAEA and S1P in C2C12 cells appears critical during myogenesis, where balanced mitochondrial activity is needed to ensure energy supply for cell differentiation.

**Figure 3:**
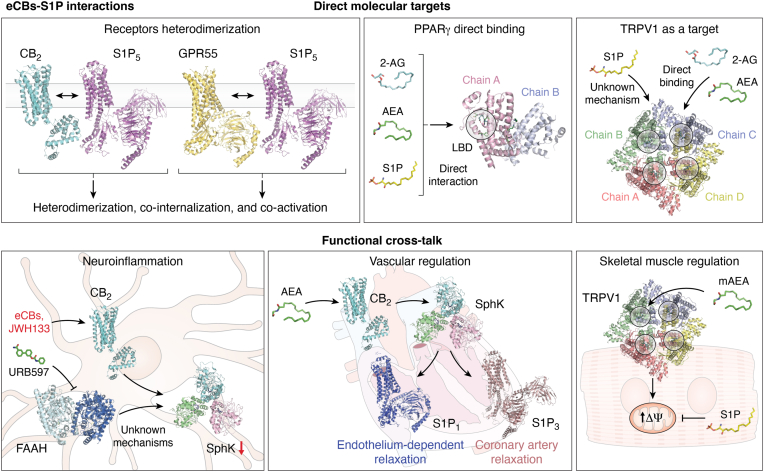
**Scheme of the crosscommunication between eCBs and S1P.** In the *upper panels*, the structural (receptor–receptor and ligand–receptor) interactions are shown. In the *lower panels* are depicted the main functional crosstalks between the two systems. The main ligand structures are represented in different colors (2-AG in *cyan*, AEA in *green*, S1P in *yellow*, mAEA in *lime*, and URB597 in *lime-green*). PDB files have been used to represent enzymes and receptor structures (CB2 in *cyan*, PDB file: 5ZTY (https://www.rcsb.org/structure/5ZTY); GPR55 in *yellow-orange*, PDB file: 8ZX4 (https://www.rcsb.org/structure/8ZX4); TRPV1 with chains in different colors, PDB file: 7LR0 (https://www.rcsb.org/structure/7LR0); S1P1 in *blue*, PDB file: 7TD3 (https://www.rcsb.org/structure/7TD3); S1P3 in *dirty violet*, PDB file: 7EW2 (https://www.rcsb.org/structure/7EW2); S1P5 in *magenta*, PDB file: 7EW1 (https://www.rcsb.org/structure/7EW1); PPARγ with chains in different colors, PDB file: 6MD4 (https://www.rcsb.org/structure/6MD4); FAAH with chains in different colors, PDB file: 3LJ7 (https://www.rcsb.org/structure/3LJ7); SphK1 with chains in different colors, PDB file: 3VZD (https://www.rcsb.org/structure/3VZD)). Image provided by Servier Medical Art (https://smart.servier.com/), licensed under CC BY 4.0 (https://creativecommons.org/licenses/by/4.0/). 2-AG, 2-arachidonoylglycerol; AEA, *N*-arachidonoylethanolamine; eCB, endocannabinoid; FAAH, fatty acid amide hydrolase; mAEA, methanandamide; PDB, Protein Data Bank; PPARγ, peroxisome proliferator–activated receptor; S1P, sphingosine-1-phosphate.

A summary of the interactions between eCB and S1P at the structural and functional levels is shown in Figure 3.

### Integrated regulation of downstream effectors

Crosscommunication between eCBs and S1P can also occur through the integrated regulation of downstream effectors triggered by the binding of eCBs and S1P to their receptor targets. Indeed, both lipid signaling systems modulate a variety of physiological processes *via* binding to G_i_-, G_q_-, and G_12/13_-coupled receptors, which are coexpressed in specific body districts (*i.e.*, CNS, immune, and vascular systems), thus allowing context-specific integration of signals. As a consequence, the cooperative modulation of effectors, like ion channels, protein kinases, and nuclear transcription factors, may have important implications in physiological and pathological processes.

Both eCB and S1P systems regulate common effectors that drive distinct signal transduction pathways, such as adenylyl cyclase, MAPK/extracellular signal–regulated kinase, PI3K/Akt, and Rho/ROCK (Rho-associated protein kinase). The crosstalk between eCBs and S1P can reinforce or weaken a specific pathway of signal transduction in both the CNS and peripheral tissues, as schematically summarized in Table 1.

**Table 1:** Commonalities in downstream effectors triggered by eCBs and S1P in different cell processes

Process	Bioactive lipid	Receptor	G protein	Effectors	Biological evidence	References
Modulation of neurotransmission	S1P	S1P_3_	G_i/0_, G_q_	↑PLC, ↑ERK	↑ Glutamate release in hippocampus, modulating synaptic transmission	(177, 178, 179)
		S1P_1_	G_i/0_, G_βγ_	↓Calcium influx	↓ Glutamate release	(180)
	eCB	CB_1_	G_i/0_	↓AC, ↓calcium mobilization, ↑MAPK inwardly rectifying potassium currents	↓ Neurotransmitter release, modulating synaptic transmission	(181, 182)
		TRPV1	NA (cation channel)	↑Calcium mobilization	↑ Synaptic plasticity and long-term depression	(183)
Immune cell trafficking	S1P	S1P_1_	G_i/0_	↑PI3K/Akt, Rac, MAPK	↑ T-cell egress from lymphoid organs	(184)
		S1P_2/3_	G_12/13_	↑Rho/ROCK	↑ Proinflammatory cytokine release in macrophages and endothelial cells	(169, 185, 186)
	eCB	CB_1_	G_i/0_	↓AC ↑MAPK	↓Cytokine release; induces an anti-inflammatory phenotype	(187)
		CB_2_	G_i/0_	↓AC ↑MAPK	↓Cytokine release modulates immune cell function and inflammation	(188)
		GPR55	G_13_	↑RhoA, Cdc42, and Rac1	↑ Cell migration and inflammatory responses	(189)
Lipid metabolism and insulin sensitivity	S1P	S1P_1-3,4_	G_i/0_, G_q_	↑PI3K/Akt, AMPK, calcium mobilization	↑ β-cell proliferation, reduce insulin resistance ↑ Liver and pancreas protection from lipotoxicity; mediates adiponectin effects in different tissues	(190, 191)
	eCB	CB_1_	G_i/0_	↑p38 MAPK; ↑ERK1/2; ↓mTORC2, ↓IRS1-PI3K-Akt ↓AMPK	↑Appetite *via* orexigenic pathways in the hypothalamus ↑ Lipogenesis and insulin resistance by reducing adiponectin levels in peripheral tissues, as liver and adipocytes	(192, 193, 194)
		CB_2_	G_i/0_, G_βγ_	PI3K activation, Akt phosphorylation ERK1/2, and CREB	↑Cell survival of β cells and metabolic regulation ↑Insulin sensitivity and reverses metabolic impairments ↑ β-cell protection	(195, 196)
Cardioprotection and vascular development/integrity	S1P	S1P_1_	G_i/0_	↑PI3K/Akt, ↑eNOS	↑ Vascular maturation and integrity	(197)
		S1P_2_	G_12/13_, G_q_	↑Rho/ROCK ↓Rac	↑ Vascular permeability and endothelial dysfunction	(2, 198)
		S1P_3_	G_13_	↑Rho/ROCK	↑Cardioprotection after I/R injury	(199)
	eCB	CB_1_	G_i/0_	↓AC, ↑MAPK	↓Cardiac contractility, associated with cirrhosis and heart failure	(200)
		CB_2_	G_i/0_	↑ PI3K/Akt, ERK1/2, STAT-3, eNOS	↑Cardioprotection after I/R injury	(201, 202, 203)

Akt, protein kinase B; AMPK, AMP-activated protein kinase; Cdc42, cell division cycle 42; CREB, cAMP response element–binding protein; eNOS, endothelial nitric oxide synthase; ERK, extracellular signal–regulated kinase; Gβγ, G beta–gamma subunits; Gi/0, inhibitory G protein alpha subunit; Gq, Gq alpha subunit; G12/13, G12 and G13 alpha subunits; I/R, ischemia/reperfusion; IRS1, insulin receptor substrate 1; MAPK, mitogen-activated protein kinase; mTORC2, mechanistic target of rapamycin complex 2; NA, not applicable; PLC, phospholipase C; Rac, Ras-related C3 botulinum toxin substrate; Rac1, Ras-related C3 botulinum toxin substrate 1; Rho, Ras homolog family member; ROCK, Rho-associated protein kinase; STAT-3, signal transducer and activator of transcription 3.

## Cannabidiol: A link between eCBs and S1P

Among the numerous constituents of cannabis (*Cannabis sativa*) preparations that interact with the eCB system, cannabidiol (CBD) has emerged as one of the most intriguing compounds for its therapeutic potential (94). The (+)-enantiomer of CBD, unlike its (−)-counterpart, has been shown to potently activate S1P_1_ and S1P_3_ receptors, thereby reducing cAMP levels in a G_i_-dependent manner (95). This effect was blocked by both dual and selective S1P_1_ and S1P_3_ antagonists, confirming the potential of (+)-CBD for the treatment of neurological disorders *via* S1P signaling (95). Notably, (+)-CBD also binds to CB_1_ as an inverse agonist (96, 97, 98), whereas (−)-CBD acts as a negative allosteric modulator of the same receptor and reduces the efficacy and potency of Δ^9^-tetrahydrocannabinol—the main psychoactive ingredient of cannabis—and AEA at CB_1_ (99). Moreover, CBD acts as a full agonist at TRPV1 channels (100), and with lower potency at CB_2_ (101) and PPARγ (102), thereby influencing anxiety, epilepsy, neuropathic pain, and inflammation (103).

In addition to eCBs—and S1P-binding receptors—CBD also modulates the metabolic enzymes of these lipids. Indeed, CBD is a potent inhibitor of FAAH, thus increasing cellular levels and signaling activity of AEA (104). CBD also modulates 2-AG metabolism by inhibiting MAGL and the other 2-AG hydrolases, ABHD6 and ABHD12 (105). In parallel, CBD influences sphingolipid metabolism by increasing S1P levels while reducing ceramide accumulation. The latter effect clearly shifts the ceramide–S1P rheostat toward cell survival and improved insulin sensitivity in the CNS (106, 107), as well as in peripheral tissues like skeletal muscles, where it ameliorates glucose metabolism (103, 106). Incidentally, another cannabis ingredient, cannabigerol, when administered in a rat model of high-fat–induced insulin resistance, increases ceramide conversion to S1P, potentially improving insulin sensitivity and providing protection from liver steatosis (108).

CBD has been shown to inhibit several CYP enzymes, particularly CYP2J2, which metabolizes eCBs into bioactive epoxyeicosatrienoic acids (109). This metabolic interference creates a complex pharmacological profile, whereby CBD not only enhances eCB tone but also redirects eCB metabolism toward alternative pathways that may influence the sphingolipid system, in particular through production of common precursors like AA.

The intersection of CBD and S1P signaling can also occur at the level of LOX isozymes, which represents an emerging and particularly fascinating area of bioactive lipid crossregulation. In fact, allosteric modulation of 5-LOX by CBD can shift its enzymatic activity from producing proinflammatory leukotrienes to generating anti-inflammatory specialized proresolving mediators (110). In line with this, S1P negatively modulates 5-LOX activity and reduces leukotriene biosynthesis to exert potent anti-inflammatory effects (83). Moreover, CBD strongly induces the formation of specialized proresolving mediators and 12/15-LOX products in resting cells by stimulating cPLA_2_-dependent release of polyunsaturated fatty acids as well as through allosteric activation of 15-LOX (110). In addition, S1P upregulates 15-LOX expression through a complex molecular pathway that engages activation of S1P_1_ and S1P_3_ receptors in macrophages, contributing to immunosuppressive tumor microenvironments (84). Overall, the intersections between CBD, S1P, and eCBs hold a promising therapeutic potential—as yet poorly explored—for the treatment of inflammatory conditions through their convergent effects on the production of bioactive lipids.

**Figure 4:**
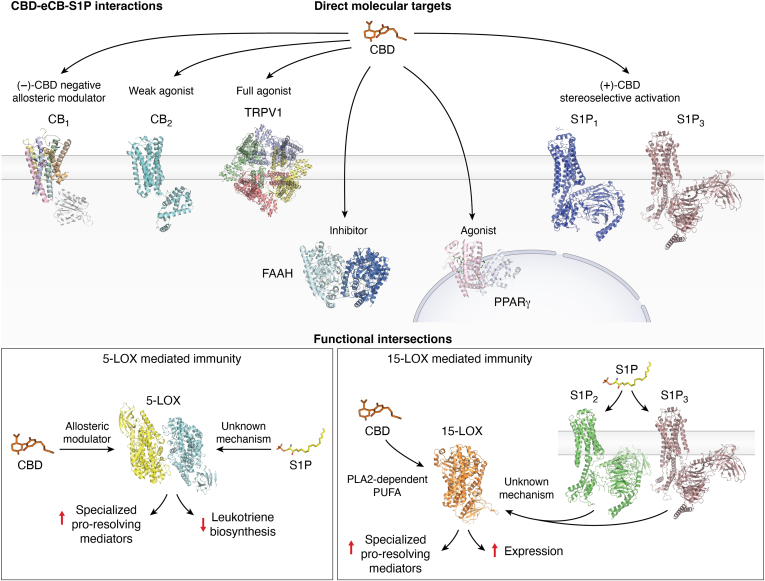
**Scheme of the crosscommunication among the CBD, eCBs, and S1P.** In the *upper panel* are reported the structural (CBD–receptor) interactions. In the *lower panels* are reported the main functional intersections exerted by CBD and S1P signaling onto 5-/15-LOX activity and expression. The main ligand structure is represented in different colors (CBD in *orange*, S1P in *yellow*). PDB files have been used to represent enzymes and receptor structures (CB_1_ with helices in different colors, PDB file: 5TGZ (https://www.rcsb.org/structure/5TGZ); CB_2_ in *cyan*, PDB file: 5ZTY (https://www.rcsb.org/structure/5ZTY); TRPV1 with chains in different colors, PDB file: 7LR0 (https://www.rcsb.org/structure/7LR0); S1P_1_ in *blue*, PDB file: 7TD3 (https://www.rcsb.org/structure/7TD3); S1P_2_ in *green*, PDB file: 7T6B (https://www.rcsb.org/structure/7T6B); S1P_3_ in *dirty violet*, PDB file: 7EW2 (https://www.rcsb.org/structure/7EW2); PPARγ with chains in different colors, PDB file: 6MD4 (https://www.rcsb.org/structure/6MD4); FAAH with chains in different colors, PDB file: 3LJ7 (https://www.rcsb.org/structure/3LJ7); 5-LOX with chains in different colors, PDB file: 7TTK (https://www.rcsb.org/structure/7TTK); 15-LOX in *orange*, PDB file: 4NRE (https://www.rcsb.org/structure/4NRE). Image provided by Servier Medical Art (https://smart.servier.com/), licensed under CC BY 4.0 (https://creativecommons.org/licenses/by/4.0/). CBD, cannabidiol; eCB, endocannabinoid; FAAH, fatty acid amide hydrolase; LOX, lipoxygenase; PPARγ, peroxisome proliferator–activated receptor γ; S1P, sphingosine-1-phosphate; TRPV1, transient receptor potential vanilloid-1.

A summary of the interactions between CBD, eCBs, and S1P at the structural and functional levels is shown in Figure 4.

## Conclusions

**Figure 5:**
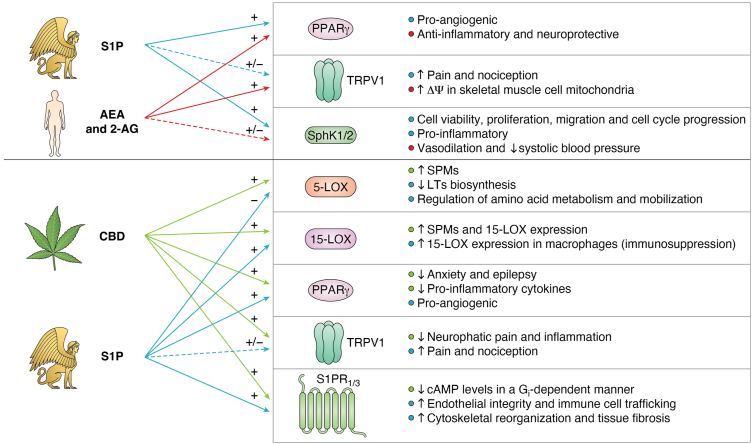
**Summary of the main common targets between eCBs, S1P, and CBD.** 2-AG, 2-arachidonoylglycerol; AEA, anandamide; CBD, cannabidiol; LOX, lipoxygenase; LT, leukotriene; ΔΨm, mitochondrial membrane potential; PPARγ, peroxisome proliferator–activated receptor γ; S1P, sphingosine 1-phosphate; S1P1/3, sphingosine-1-phosphate receptor 1/3; SphK1/2, sphingosine kinase 1/2; SPM, specialized proresolving mediator; TRPV1, transient receptor potential vanilloid 1. Image provided by Servier Medical Art (https://smart.servier.com/), licensed under CC BY 4.0 (https://creativecommons.org/licenses/by/4.0/).

This review has highlighted how eCBs and S1P represent two complex signaling networks that, beyond canonical GPCR activation, can mutually influence metabolism, transport, trafficking, and transcription factor activity of each other. Both systems interact through multiple coregulatory mechanisms, such as receptor heterodimerization and coregulation (*e.g.*, S1P_5_, CB_2_, GPR55, PPARγ), shared metabolic enzymes (*e.g.*, SphK1–2, LOXs), and integrated downstream effectors, particularly of TRPV1, as summarized in Figure 5. This interplay results in finely tuned, context-dependent modulation of bioactive lipids in cellular responses, with an impact on the regulation of inflammation, vascular function, neuronal activity, and insulin sensitivity.

Furthermore, the cannabis ingredient CBD demonstrates significant potential as a modulator of eCB and S1P endogenous systems. By modulating dioxygenase enzymes that metabolize eCBs and S1P—such as LOX, COX, and CYP isoforms—CBD further underscores the biochemical convergence of their pathways. Therefore, the activity of these enzymes represents a metabolic “checkpoint,” where the outputs of eCB and S1P signaling, along with those of CBD, are integrated and finely tuned.

On a final note, recent studies have revealed novel mechanisms of crosscommunication between bioactive lipid systems. In fact, an intriguing discovery has shown that the nuclear translocation of a truncated form of S1P_2_ induces PPARα degradation, which, as a consequence, blocks transcription of lipid catabolic genes (111). Moreover, a novel regulatory role of NAPE-PLD as an unexpected target of thiazide diuretics has been recently reported, pointing at it as a moonlighting enzyme (112). These findings further support the notion that our current understanding of lipid signaling is still in its infancy, and so far, only the tip of the iceberg has been scratched.

In conclusion, it seems apparent that eCB and S1P signaling pathways operate through interconnected networks of remarkable complexity. As yet, the biochemical crosstalk between these bioactive lipids remains incompletely understood, potentially limiting the therapeutic exploitation of these signals. Future strategies targeting the spatiotemporal dynamics of lipid transport—from intracellular trafficking to extracellular distribution—combined with selective receptor engagement may unlock novel therapeutic opportunities that current approaches have not fully realized.

## Conflict of interest

The authors declare that they have no conflicts of interest with the contents of this article.
